# Intersexuality/Differences of Sex Development through the Discourse of Intersex People, Their Relatives, and Health Experts: A Descriptive Qualitative Study

**DOI:** 10.3390/healthcare10040671

**Published:** 2022-04-02

**Authors:** María de las Nieves Moreno-Begines, Almudena Arroyo-Rodríguez, Álvaro Borrallo-Riego, María Dolores Guerra-Martín

**Affiliations:** 1 San Luis Health Center, Andalusian Health Service, 41003 Seville, Spain; marianieves.moreno1995@gmail.com; 2 University Nursing Center “San Juan de Dios”, University of Seville, 41930 Seville, Spain; almudena.arroyo@sjd.es; 3 Faculty of Nursing, Physiotherapy and Podiatry, University of Seville, 41009 Seville, Spain; aborrallo@us.es

**Keywords:** attitude, disorders of sex development, intersex persons, qualitative research

## Abstract

Intersex/differences of sex development (DSD) conditions are divergences among genitalia, gonads, and chromosome patterns. These variances have been present for millennia and socially defined according to the cultural system established. The aim of this study is to describe the perspectives of adult intersex/DSD people, their relatives, and intersex/DSD expert professionals in Spain. A descriptive qualitative study design was adopted. The study was carried out in several locations in Spain. Individual in-depth interviews were conducted and addressed to 12 participants (4 intersex/DSD people, 3 relatives, and 5 professional experts). A total of 4 spheres, 10 categories, and 26 subcategories were obtained. The number of verbatims obtained in each of the spheres described were intersex/DSD as a community (*n* = 54), health sphere approach (*n* = 77), law sphere approach (*n* = 12), and psychosocial approach (*n* = 73). Regarding intersex/DSD as a community sphere, there is a clear need of promoting education on sex and body diversity. With respect to the health sphere, it is mentioned the inadequacy of services and how this has a negative impact on the health of intersex/DSD people. Regarding the law sphere, it is highlighted the need of designing legislations at a national level which protect and defend the rights of intersex/DSD people. Regarding the psychosocial sphere, these people suffer from social isolation, secrecy, shame, self-identity questioning, and mental disorders that negatively impact their quality of life.

## 1. Introduction

Intersex/differences of sex development (DSD) variations involve the presence of discordances among the chromosomal, gonadal, and phenotypic sex determination criteria. These are originated during the process of sex determination in the foetal life, and involves multiple factors such as genetic variance, or secondary factors related to maternal or environmental influence, affecting both gonadal and genital development and originating ambiguity in sex genitalia [1,2]. Intersex people are born with sexual characteristics that generally do not fit the typical definitions of male and female. For this reason, in many countries, children are subjected to surgeries or other early health interventions with the intention of normalizing their bodies [3]. In this regard, the study by Agramonte et al. [4] identified that 50% of intersex/DSD people abandoned the health services due to disappointment and frustration in view of the results of surgeries and attention received. According to Lorenzo et al. [5], 50% of parents of intersex/DSD minors expressed regretting their decision on early surgery.

Although the management of DSD remains controversial, as many experts consider bodily variations a manifestation of the diversity of the human being individualized and multidisciplinary care is necessary [3,6].

In relation to the incidence of intersex/DSD conditions, there can be observed a great complexity and discrepancy among the number of cases. According to Woodward and Burns [7], this rate occurs in 1 or 2 cases out of every 10,000 live births. Notwithstanding, the most widespread and recognized data was provided by biologist Fausto-Sterlin [8], who established the prevalence between 5 and 170 cases per 10,000 live births in the world.

In the international sphere, different legislations related to legal recognition of intersex people, as it is the case of Australia in March 2010, and Germany in 2013, allow the selection of a third choice for gender on birth certificates and passports. However, Spanish law requires the registration of newborns in one of the two sexes in the Civil Registry (within 1–8 days, extendable up to 30 days) [9,10].

Regarding international policies, several countries have different laws which prohibit, restrict, or condemn non-consensual surgeries and treatments to intersex/DSD minors, as it is the case of Malta and Chile, which passed these legislations in 2015, and Portugal in 2018. However, in Spain there are no laws for the time being in force relating to the rights of intersex/DSD people, but there are regional policies in 7 of the 17 autonomous communities in the country [9,11]. In this sense, laws have been proposed to ensure the rights of intersex people, as well as to guarantee social equality and non-discrimination based on gender identity, gender expression, and sexual characteristics [12,13].

Mental healthcare for children and parents can be highly helpful but may also be interpreted by a child as a message that something is wrong with them [14]. Bennecke et al. [15] identified that 40.4% of parents with intersex children needed psychological support. However, the effect of psychosocial interventions is hardly investigated [3].

Regarding intersexuality as part of the LGBT community, we must point out that in 2009, it was accepted to incorporate intersexuality, adopting the LGBTI sequence. However, not all intersex people agreed with that inclusion [10].

The objective of the study is to describe the perceptions of intersex/DSD people, their relatives, and professional experts.

## 2. Methods

### 2.1. Design

A descriptive qualitative research design was used, and data were collected from February 2020 to November 2020. Such an approach can offer a rich description of a phenomenon about which little is known [16]. Furthermore, the goal of a qualitative descriptive study is to obtain knowledge of the experiences, events, and interactions of a phenomenon from the viewpoint of the insiders [17]. Because the goal of this study is to describe the perceptions of intersex/DSD people, their relatives, and professional experts in the field, a qualitative descriptive design was particularly relevant.

### 2.2. Participants

A convenience sampling strategy was used to recruit 12 participants from several locations in Spain. Such a sampling strategy allows researchers to identify information-rich individuals within the phenomenon of interest by the virtue of their knowledge and experience [18]. This strategy can facilitate an in-depth understanding of the research area [19].

The inclusion criteria were: In profile 1, intersex/DSD people: (a) age 18 or older, and (b) in one of the intersex/DSD categories according to the 2006 Chicago declaration [20]. In profile 2, relatives of intersex/DSD people: (a) father or mother of an intersex/DSD person, and (b) have lived together or collaborated in the health process for more than 6 years. In profile 3, expert professionals in intersexuality/DSD: (a) collaborate as a professional (doctor, nurse, jurist, psychologist, and anthropologist) in an association related to care for intersexuality/DSD, and (b) carry out their research work or care regarding intersexuality in Spain. The exclusion criterion in the three profiles was not granting consent to participate.

### 2.3. Data Collection

A total of 12 individual in-depth interviews were used for data collection. Interviews were conducted by the first author online, via Skype^®^ and Zoom^®^, as well as by phone. The investigators first screened for potential research participants according to the eligibility criteria. Then, the first author approached potential participants and provided to them a comprehensive explanation of the study details. Interested potential participants were then each issued an information sheet and consent form. Confidentiality and anonymity were ensured. A code was assigned to each participant to safeguard data confidentiality and anonymity.

As a result of the COVID-19 pandemic, participants were at home during these interviews and in the session only interviewer and participant were present. The interviews were audio-recorded and later transcribed verbatim. Data were collected from July to November 2020.

### 2.4. Data Analysis

Each interview was transcribed verbatim by the researchers. Thematic analysis was adopted to identify, analyse, and report themes within the data. The researchers analysed the data in three stages: data familiarising, coding, and data reduction [21]. Data analysis was conducted concurrently with the data collection to allow data familiarising [22]. Basic elements related to the phenomenon were extracted to identify initial codes and were categorised [21]. Initial codes were explored before grouping them to form a subcategory. The subcategories were then examined and grouped into potential categories. Subsequently, the categories were explored and grouped into potential spheres. Two researchers (MN M-B and A A-R) independently reviewed the transcripts to derive the initial categories and subcategories. Regular meetings were held to discuss and triangulate the final categories and subcategories. Any discrepancies between the two authors were clarified by involvement of a third author (MD G-M). The spheres, categories, and subcategories are shown in Supplementary Materials Table S1.

Credibility was achieved through familiarization, triangulation, and peer review of the collected data. Triangulation was achieved by crosschecking data such as audio-recordings and transcripts among researchers.

### 2.5. Ethical Approval

Ethical approval for the study was granted by a regional research ethics committee (approval: 1421-N-20). Informed consent was obtained from all participants prior to the interview. Participants were free to withdraw from the study at any time.

## 3. Results

A total of 12 participants were part of this study: intersex/DSD people (*n* = 4), intersex/DSD people’s relatives (*n* = 3), and professional experts in intersex/DSD (*n* = 5). The dominant sex was women (*n* = 10) versus men (*n* = 2). An amount of 4 spheres, 10 categories, and 26 subcategories were obtained. In addition, suggestions for improvement were obtained. The results obtained in the study are presented below. The number of verbatim extracted from the interviews are shown in Table S1.

### 3.1. Sphere 1: Intersex/DSD as a Community

#### 3.1.1. Category: Intersex/DSD Term

##### Subcategory: Knowledge about the Term Intersex/DSD

Regarding this subcategory, it can be observed how both intersex/DSD people and their families manifested unawareness about the existence of this term and its meaning prior to being acquainted with this reality. The same situation applies to professional experts, who declared the same unawareness or even receiving scarce information during their education. In this regard, they expressed the need for more training and knowledge about the realities of intersex/DSD people.

F_HSC: “[…] I mean… I was not familiar with it at all, I knew nothing about those terms”.

F_SIA2: “[…] Doctor told us about all this, it was a shock, I mean, we didn’t know what this was about”.

##### Subcategory: Identity with Respect to the Term Intersex/DSD

There are some inconsistencies in relation to the sense of identity on the part of participants towards the different terminology. It can be observed that both people with androgen insensitivity syndrome and their relatives associate the term DSD to negative and pathological connotations, whereas the term intersex was considered positive. In addition, they manifested to have a sexual anatomy-related condition, not a disease. However, the study identifies that the profiles diagnosed with Klinefelter and Turner syndromes and congenital adrenal hyperplasia preferred to be identified with a clear diagnostic label and considered that they had a pathology. Notwithstanding, the professional experts highlighted the inadequacy of the term DSD to be used in a wide sense due to the pathological burden implied.

A_SIA: “[…] I prefer the term intersex conditions, in plural. I normally use it in the plural form, so people understand that there are different and diverse realities related to intersex conditions […]”.

F_HSC: “[…] I didn’t like the term intersex. The truth is I didn’t like it because it seems to refer to something weird. It seems that you are weird”.

F_SIA1: “DSD is a medical, pathological term which comprises different variations of sex characteristics as malformations […]”.

##### Subcategory: Intersex/DSD as a Whole

There were discrepancies between people with these conditions and expert professionals on the adequacy of the umbrella term intersex/DSD to include all existing realities. However, there was consensus in the discourse of relatives, who accepted the use of the term intersex referring to all these conditions as a whole because it may help to promote their visibility in society.

A_HSC: “[…] Yes, I think we all have a lot in common, all intersex people. So, yes, I think it is correct to gather… I think it is also a way to create bonds”.

F_HSC: “[…] I can see the differences, but I also think that they are all related, so we are all part of the same reality”.

F_SIA2: “[…] Part of society still does not consider it normal, so I would agree to be included into this group. I don’t know if I’m making myself clear… As a mother, I see my daughter as normal, I don’t need her to be part of whatever group because, to me, she is my daughter, and… she is perfect the way she is”.

##### Subcategory: Intersex/DSD as Part of the LGBT Community

Regarding the needs and adequacy of the term intersex/DSD within the reality of the LGBT community, some discrepancies were observed. Some people highlight that the LGBT reality relates to identity experiences and sexual orientation, whereas the reality of intersex/DSD people is more connected to biological aspects. However, it is also noticed how advocates claim common experiences and necessities in both communities, which would favour stronger social visibility. Concerning the perspective of both relatives and expert professionals, some discrepancies are also observed due to what some of them consider a powerful tool to bring visibility to intersex/DSD, if this is based on a real presence of this reality within the community. On the contrary, other interviewees consider that the demands are different and must be achieved separately.

A_ST: “I think this is something completely different, maybe the problem area can be a little similar, but I don’t see it that way”.

A_SIA: “Well… at the beginning… when it was included, it seemed weird to me, right? Because I didn’t feel as part of the community, as this is not like a sexual orientation or gender identity […] But I think that the inclusion brings a lot of visibility. I also realize that we have similar experiences […] so I feel very comfortable now […]”.

E_A: “[…] I think intersexuality has nothing to do with sexual orientation or gen-der identity, but it is a body diversity which it is related to sex, so it could be adequately included there”.

#### 3.1.2. Category: Organizations

##### Subcategory: Organizations Benefits

In relation to the discourses held by the different profiles, it is observed some consensus in the answers provided, which described how organizations bring benefits at a psychosocial level. Speeches highlighted how this resource fosters the sense of belonging to the group, brings social visibility, and provides support and guidance in the healthcare process. However, interviewees pointed out the small number of organizations in Spain.

A_SIA: “[…] It is a way of feeling included, being a part of a group, part of society, and that little by little barriers begin to be removed, and oppositions and fears start to disappear”.

F_HSC: “[…] It has helped me a lot because… well […] It is truly a backing. It is a strong support, comfort, and… well… sometimes even a room for small consultation, though not strictly to health professionals […]”.

E_J: “In the case of Spain, it is complex because there are just a few”.

##### Subcategory: Contact between Organizations of Different Types of Intersex/DSD Conditions

The participants rarely knew the syndromes included in the different intersex/DSD realities, nor their connections. There is little contact between the organizations of the different types of intersex/DSD conditions.

A_SK: “I was in contact with FEDER prior to create the association […] I also talked with Genes y Gentes foundation […]”.

E_PSIA: “[…] He has 5-alpha reductase deficiency, one type of intersex conditions, and I have contacted him”.

### 3.2. Sphere 2: Health Sphere Approach

#### 3.2.1. Category: Communication of Diagnosis

##### Subcategory: Diagnosis Detection

In relation to intersex/DSD people, it is observed how the majority did not know their full diagnosis and how the discovery happened in adult life, due to the secrecy in family circles.

A_SK: “[…] I had my diagnosis when I was 25 […] they discarded leukocytosis and… they consulted an endocrinologist who asked for a series of tests and told me that the fatigue could be gonadal-related. From there, hypogonadism and then, I don’t know why the hell… she decided to perform a karyotype, which resulted in a SK”.

A_SIA: “[…] I was 21 […] I didn’t have the procedure they recommended… the vaginoplasty, basically because I was afraid. Then I went back to the gynaecologist, and it was then when I was fully informed […]”.

##### Subcategory: Healthcare

With respect to intersex/DSD people and their families, it should be highlighted the lack of sensitivity and empathy on the part of health professionals in the diagnosis communication, as well as the absence of clear explanations regarding the physical implications involved in the different diagnoses.

A_ST: “[…] Doctors only told me it was a hormone disorder, and that was all”.

F_SIA2: “[…] Looking back, everything was a bit catastrophic then”.

#### 3.2.2. Category: Health Process

##### Subcategory: Risk Behaviours

It is observed how intersex/DSD people show risk behaviours (suicidal tendencies, compulsive sex drive, etc.), as well as mental disorders. However, these conducts are not directly associated with their condition, but they are rather the result of the social pressure experienced by the need of belonging to one of the two genders (man/woman) defined by the heteronormative social system.

E_PSIA: “[…] My compulsive drive was to have constant sexual intercourse, nymphomaniac? No, I didn’t feel pleasure, I just needed to reaffirm my feminine self”.

##### Subcategory: Surgical Interventions

Several of the intersex/DSD people participating in this study underwent genital surgical procedure, either gonadal or vaginoplasty. It is also observed that there are discrepancies in this profile and their relatives towards the defence or criminalization of these procedures in intersex/DSD minors. Detractors of surgical interventions point out the different negative consequences these procedures cause at physical and psychological levels. However, there is agreement among expert professionals, who strongly disapprove of minors undergoing surgical procedures without their own consent, as well as of the social implication in determining the application of these procedures.

A_SIA: “[…] The question of gonadectomy is really serious, as well… at least in my case, because, of course, it has huge physical and psychological consequences”.

F_HSC: “[…] I am positive I would do the same over and over, it is fantastic”.

E_A: “[…] If it not consented to, when you are a baby and the like, I’m fully against. To me that is maiming and abuse. A total neglection of this person’s rights”.

##### Subcategory: Treatment and Examinations

Regarding treatments and examinations in the health process, it is observed how the different profiles of participants denounce the degrading and humiliating treatment on the part of health professionals and indicate the infringement of fundamental right to privacy as well as the lack of medical monitoring during examinations.

A_SIA: “[…] from examinations in which all the health professionals, all the gy-naecologists had to be there… students, rights? It’s outrageously humiliating and abusive […]”.

F_SIA2: “[…] They wanted to experiment with our daughter, because the options we were given, they were all awful”.

##### Subcategory: Limitations of Healthcare Benefits

There is unanimity in the discourse of the different profiles when denouncing the lack of information among health professionals in relation to the intersex/DSD conditions, as well as the lack of updated treatments and discrimination in healthcare.

A_SK: “[…] Most of the times, when I went to a doctor, I swear, they literally told me: “I studied this on my first year”. And then they googled it, right in front of me! Then is when you feel absolutely abandoned, like very… very… untended […]”.

F_HSC: “[…] lack of knowledge on the part of nurses, on the part of those who keep medical histories… in this sense… feh… [sighs] really, really, really forsaken […] drug coverage, in the case of chronic patients… it should be covered by the hospital and not a magistral medicine which I have to order at the pharmacy and therefore subject to withholding taxes applied to me […]”.

E_A: “[…] The biggest problem area professionals find in these cases is lack of knowledge […]”.

### 3.3. Sphere 3: Law Sphere Approach

#### 3.3.1. Category: Strengths/Weaknesses of Policies against LGBT Discrimination

##### Subcategory: Legislations Concerning ‘Gender Normalizing’ Surgeries for the Intersex/DSD Population

Some discrepancies are observed in the participants’ discourses. Some of them denounce the immobility and non-compliance of the Spanish government with regards to surgeries performed on minors, whereas other participants reject those legislations.

A_SIA: “[…] Why forcing a surgery on a child when there is nothing wrong to fix? You know what I mean? When there is no health issue and even when this health issues are going to appear precisely after the intervention, right? Affecting both physical and psychological health. Of course, this should be regulated because who if not is protecting them?”.

F_HSC: “[…] I think it is an abomination to ban surgeries until adult stages and of course that these procedures are eliminated from the healthcare system […]”.

##### Subcategory: Policies on the Undefined Sex

Regarding the policies on the existence of a third gender category on health and law records besides man and woman, intersex/DSD people do not endorse the initiative, as they consider it a new way of classifying and discriminating their realities. However, among expert professionals, some discrepancies are observed in terms of the adequacy of implementing this category, as some professionals advocate for its erasure and others consider it a useful tool.

A_SIA: “[…] There is a lot of social pressure and law urge to register a baby, when this is totally unnecessary”.

E_E: “[…] Societies in which this is so well done, in which they have designed or included other checkboxes […] people who are other thing, nor men or women, and they are called something different… and there some others can fit, people who in a natural, nor artificial way, are born with a given sex but they do not feel comfortable with the gender in which that sex normally develops in our society […]”.

### 3.4. Sphere 4: Psychosocial Approach

#### 3.4.1. Category: Social Relations

##### Subcategory: Friends

It can be observed that there are different synergies among the discourses of intersex/DSD people, who declare to have found difficulties in establishing social relations, or even that they experimented different types of bullying, discrimination, or abuse.

A_SK: “[…] Yes, I spent 14 years without a single friend, and that caused me… well… not a single friend because I was bullied at school […]”.

A_SIA: “But the first years were of complete isolation, it is exactly that, isolation […]”.

##### Subcategory: Relatives

Intersex/DSD people declare to currently have or have had periods of difficult relations with their relatives. This is due to the stress situation within the family.

A_SIA: “It affected us all very deeply. I think any of us couldn’t cope or manage. My mother didn’t know how… and… well, I think that my family used to stuck away all what was overwhelming, to make as if something didn’t exist, so maybe it would go away […]”.

A_HSC: “[…] the relationship with your parents, well I speak for myself, in my own experience, it was awful, really, one of the biggest problems I’m facing now is to forgive [quotation marks] what I blame them for, because, on top of that, we had family conflicts when living together”.

##### Subcategory: Partners

Regarding sex life and relationships, it is observed that it is one of intersex/DSD people’s major concerns. The rejection of their condition and low self-esteem, produce difficulties in establishing couple relationships.

A_SK: “[…] But I have experienced that before getting married and being with girls… Having a coffee or whatever, telling any girl my story… and they simply dis-appeared, just like that. It’s hard… really… really hard […]”.

F_SIA2: “[…] Then we clashed sometimes… there were frictions when I started to get angry with my doctor and I didn’t want him anymore”.

#### 3.4.2. Category: Daily Dynamics

##### Subcategory: Work

Some synergies can be observed among the discourses of the different participant profiles, which indicate how intersex/DSD involves limitations in the work dynamics.

A_HSC: “[…] I was making some slips in the professional field, I started to have problems, became a recluse sort of woman”.

F_HSC: “[…] It means a lot of boundaries in your life, your work, I had to schedule and organize drug intakes… I travel a lot… I lead a department… so, at a professional level… phew… that’s crazy”.

##### Subcategory: Family

Similar discourses are identified among the different profiles of participants, expressing how intersexuality/DSD creates a climate of secrecy that prevents sharing their condition with relatives, which affects family dynamics.

F_HSC: “[…] It was extremely hard, a lot of money, dedication and struggling… Hmm… I don’t know how to explain it… a lot of effort”.

#### 3.4.3. Category: Psychological Impact

##### Subcategory: Feelings after Diagnosis Communication

Similar feelings are identified among the different participant profiles. It is observed disbelief and even difficulties in processing the information. With respect to their relatives, they experience shock situations and even rejection of this reality.

F_HSC: “[…] Everything fell apart […] it was horrible, the first days were a huge shock […] at the beginning, I don’t know… even rejected the girl, a little”.

F_SIA2: “[…] It was a tremendous blow […] Then, damn… it was like beating one’s head against the wall […]”.

##### Subcategory: Self-Perception

All intersex/DSD people interviewed showed a similar discourse and indicated to have experienced low self-esteem, rejection, and shame towards their own bodies and may suffer from dissociative disorders to cope with the stress of having to fit into the man/woman dichotomy culturally imposed.

A_SK: “[…] it was… a really hard process, really, a process… of learning and acceptance in the first place […]”.

A_SIA: “[…] there were like two versions of myself, an external one who pretended to be happy, as I was a normal woman, right? As it should be, and then, internally, I was someone who trusted no one, feeling ashamed of myself, and feeling deeply rejected […]”.

#### 3.4.4. Category: Social Influence

##### Subcategory: Social Unawareness

It is observed how the discourses of both intersex/DSD people and their families denounce the lack of knowledge and visibility of these conditions in society, the latter sometimes encouraged by health professionals who argue that the outreach could also prompt a backlash against the emotional stability of people with these conditions. However, this scenario creates situations of social isolation.

A_HSC: “[…] 98% of the population is completely unaware or they have no idea of it”.

E_PSIA: “[…] I have a strong phobia of someone finding out that”.

FAMSIA2: “[…] The first thing the doctor said was that we have to hide it and not telling family or friends about it, not telling anyone because it may cause harm. Every-thing was about hiding and hiding […] We’ve been hiding it all this time”.

##### Subcategory: Influence of Gender Stereotypes

In the discourses of intersex/DSD people, it can be also identified how they suffer besides symptomatology and pathologies associated to their conditions—a denial of their masculinity and femininity.

A_SIA: “[…] what I felt is that, if I revealed my secret, it would be impossible for me to be with a man, because I considered myself heterosexual, ok? … so, accepting me without having a functional vagina, mmh… I lived like that and that was the only option I had all those years […] but it seems to me that what women’s pleasure or desire don’t matter, right? The only thing that matters is to be normal and able to satisfy… to be a penetrable body […]”.

A_SK: “[…] Immediately, most of the population links inability to reproduce with sexual incapacity or non-performance. So, in the end they say: “This guy is a eunuch”, and it has happened to me, they have said that to my face and it is very hard […]”.

### 3.5. Suggested Improvements

From different profiles, the suggested improvements in the care approach include the urgency of having multidisciplinary teams, educational campaigns for health professionals, and larger number of services.

It is observed in the different discourses the need of promoting measures to foster social visibility and enlarging the presence of psychologists and mental health professionals in the health approach.

Participants declare their need and aspiration to implement legislation in Spain related to the rights of intersex people, with the aim of guaranteeing sanctions for actions that violate the rights of people, such as surgical interventions on intersex/DSD minors.

A_ST: “[…] It will be very interesting to have a multidisciplinary team which included a mental health team […]”.

E_A: “[…] In my opinion there is a huge lack of health protocols after diagnosis and a lot to be done about terminology for each specific case […]”.

E_E: “[…] It is necessary to train… nurses, doctors… in the treatment, in concepts that, after all, are social constructs, so we can dispel prejudices, right? Also, regarding patient care […]”.

A_SK: “[…] Everything should be more supportive… culturally, socially, educationally, and from a health perspective so… so we can be part of society […]”.

F_SIA1: “Social and educational visibility of the existence of body/genital sex characteristics different from the extreme masculine-feminine”.

E_J: “[…] Hence the importance of providing visibilization of intersex people, pointing out that still today we are consenting to serious violations of human rights in the case of intersex people […]”.

F_SIA1: “The reality of intersex people does not exist in the national registers by law […] it must exist in the register law of this reality”.

## 4. Discussion

This study detects a lack of information in the different groups. In this sense, García-Dauder et al. [23] stated that information addressed not only to those affected and their relatives, but also to medical professionals, is very important. As in the study by Gregori [24], it can be observed how data obtained show similarities in the sense of indicating participants’ lack of knowledge prior to their immersion in the intersexual/DSD reality. In addition, there are also discrepancies with respect to the inclusion of intersex/DSD people within the LGBT community. In addition, different discourses regarding the adequate term usage can be observed, as well as the need of a global terminology to identify the different conditions and their consideration as a pathology on the part of the different profiles, which is sustained by the data obtained in the studies by Lundberg, Hegarty, and Roen [25] and Gregori, García, and Hurtado [26].

There are some synergies with the study by Machado et al. [27], which indicated how the ideal model is the one focusing on the patient which provides a holistic approach, while fostering a cooperative relationship doctor-patient where the latter enjoys autonomy. In addition, professionals must guarantee an updated, expert knowledge of this subject and provide complete, clear information, advice, and the will of contributing to an appropriate referral and monitoring [9,15,28].

There is a social and family secrecy about intersex/DSD people, which causes late diagnosis detection. With respect to healthcare, it is highlighted the lack of sensitivity and empathy on the part of health professionals in the communication of diagnosis, their lack of knowledge regarding these realities, the scarce number of multidisciplinary teams, and the absence of an adequate healthcare process, which is currently marked by lack of intimacy and exhibitions during medical examinations and other considered of interest in terms of psychological health. These findings are consistent with the results obtained in the studies by Gregori [28] and Gregori, García, and Hurtado [26]. Because of this scenario, this population exhibits more health risk behaviours [29].

In addition, some intersex/DSD people relatives express regret after applying surgical procedures, which coincides with other studies such as those by Lorenzo et al. [5] and Roen, Creighton, Hegarty, and Liao [30]. On the other hand, some participants defend these surgical procedures, stating the need of these practices to guarantee an adequate social integration of the minor and avoid a negative psychological impact. This coincides with other authors such as Witchel [31]. According to Inter, Bauer, and Truffer [11], it is patent how the criteria established in the Chicago Consensus 2006 are ignored and early surgery is performed on minors, violating their rights. Participants point out different proposals to foster social visibility of intersex/DSD conditions such as educative workshops on sex and body diversity, and the introduction of these subjects in the health professionals’ academic field, schools, and colleges, thus avoiding a pathologizing approach and advocating the necessity of implementing legislations on behalf of intersex/DSD people’s rights in Spain, as well as introducing sanctions [9,15,28].

After birth, families of babies with ambiguous genitalia experience stress towards the obligation of registering the baby within the dichotomous classification of sex in the Spanish Civil Registry. However, as the studies by Gregori [24] and Lauroba [10] pointed out, most of intersex/DSD population advocate for the elimination of the sex/gender box.

Regarding the social sphere, it is revealed how the need of hiding and the feeling of guilt on the part of relatives towards intersex/DSD people produce a negative impact on family relations. In addition, intersex/DSD people may suffer from different forms of bullying, discrimination, or abuse, as well as difficulties to establish social or partner relationships. This is mainly due to the fear intersex/DSD people experience towards the rejection of their condition and the presence of low self-esteem, body shame, and rejection, and even dissociative disorders when facing the need of belonging to the woman/man dichotomy established by rigid, heteronormative systems that reject body diversity and constantly depreciate intersex/DSD people’s femininity/masculinity. All this pressure leads to the need of constantly pursuing the established standards of what is considered man or woman [24,32,33].

Additionally, it is observed how the presence of isolation behaviours and mental disorders associated to the different syndromes may cause alterations in the work dynamics of intersex/DSD people [29,34].

With respect to their families, their members may present a major psychological impact after the diagnosis of the intersex/DSD condition, resulting in the lack of free time and disruption in family and work dynamics [35].

The need of providing psychological support to build self-esteem, eliminate stigmatizing images, and redefine the notion of normality is observed [9,15,28].

### Limitations

Phenomenology intrudes on people’s private worlds, so trust can be a limiting factor, especially if participants are talking about sensitive information [36].

As a result of the COVID-19 pandemic, interviews were conducted online and over the phone, making it difficult to interact with the other person, which would be more possible in a face-to-face interview.

## 5. Conclusions

Regarding intersex/DSD as a community field, there is a clear need to promote information and education on sexual and bodily diversity. The inclusion of intersex/DSD people within the LGBT community presents some discrepancies among the participants, since not all are represented in it.

With respect to the health sphere, there is a late detection of the diagnosis due to family secrecy. Professionals are required to have adequate training and awareness to care for intersex/DSD people. A holistic, patient-centered approach is needed to decrease health risk behaviors. Discrepancies are detected regarding the suitability of surgical procedures.

In the law sphere, the need to design legislation in Spain that protects and defends the rights of intersex/DSD people are highlighted, thus complying with the criteria established by international organizations.

Regarding the psychosocial sphere, family relationships are affected by the feeling of guilt of relatives. Likewise, couple, work, and social relationships are affected by the harassment, discrimination, or abuse that these people may suffer. The strict social norms related to male/female stereotypes have a negative influence. Intersex/DSD people experience social isolation, secrecy, shame, questioning of their own identity, and mental disorders that diminish their quality of life. It is necessary to provide adequate psychological support. This study has contributed to identify relevant aspects about the approach and influence of intersex/DSD in the different profiles involved. These findings can be used as a foundation in future studies which may establish prospective research areas while visibilizing the realities and demands of intersex/DSD people among health professionals, with the aim of improving the healthcare provided to these people and their families.

## Supplementary Materials

The following supporting information can be downloaded at: https://www.mdpi.com/article/10.3390/healthcare10040671/s1, Table S1: Spheres, categories, subcategories, and number of verbatims extracted from the interviews.
