# Supplementary material for: Intersexuality/Differences of Sex Development through the Discourse of Intersex People, Their Relatives, and Health Experts: A Descriptive Qualitative Study
Source: Healthcare (Basel). 2022 Apr 2;10(4):671. doi: 10.3390/healthcare10040671 (PMC9032209; doi:10.3390/healthcare10040671)
Supplement: Supplementary file 1 [file healthcare-10-00671-s001.zip › healthcare-1619065-supplementary.pdf]

Table S1. Spheres, categories, subcategories and number of verbatims extracted from interviews

Spheres	Categories	Subcategories	Verbatim (N)
Intersex/DSD as a Community	Intersex/DSD Term	- Knowledge about the term Intersex/DSD	N=3
		- Identity with respect to the term Intersexl/DSDion	N=8
		- Intersex/DSD as a Whole	N=9
		- Intersex/DSD as part of the LGTBIQ+ community	N=13
	Organizations	- Organizations Benefits	N=12
		- Contact between Organizations of Different Types of Intersex/DSD	N=9
Health Sphere Approach	Communication of the Diagnosis	- Diagnosis detection	N=8
		- Healthcare	N=10
	Health Process	- Risk behaviors	N=2
		- Surgical interventions	N=22
		- Treatments y Examinations	N=10
		- Limitations of Healthcare benefits	N=25
Law Sphere Approach	Strengths/ Weaknesses of the Policies Against LGTBIQ+ Discrimination	- Legislations Concerning 'gender normalizing' surgeries for the Intersex/DSD population	N=8
		- Policies on the Undefined Sex	N=4
Psychosocial Approach	Social Relations	- Friends	N=6
		- Relatives	N=3
		- Partners	N=9
	Daily Dynamics	- Work	N=5
		- Family	N=5
	Psychological Impact	- Feelings after Diagnosis Communication	N=5
		- Self-perception	N=9
	Social Influence	- Social Unawareness	N=18
		- Influence of Gender Stereotypes	N=13
